# Potentials, Challenges, and Genetic and Genomic Resources for Sugarcane Biomass Improvement

**DOI:** 10.3389/fpls.2018.00151

**Published:** 2018-02-16

**Authors:** Ramkrishna Kandel, Xiping Yang, Jian Song, Jianping Wang

**Affiliations:** ^1^Agronomy Department, University of Florida, Gainesville, FL, United States; ^2^Horticultural Sciences Department, University of Florida, Gainesville, FL, United States; ^3^College of Life Sciences, Dezhou University, Dezhou, China; ^4^FAFU and UIUC-SIB Joint Center for Genomics and Biotechnology, Fujian Provincial Key Laboratory of Haixia Applied Plant Systems, Fujian Agriculture and Forestry University, Fuzhou, China

**Keywords:** biomass, sugarcane, energy cane, cell wall databases, biomass candidate genes, second-generation biofuel

## Abstract

Lignocellulosic biomass has become an emerging feedstock for second-generation bioethanol production. Sugarcane (*Saccharum* spp. hybrids), a very efficient perennial C4 plant with a high polyploid level and complex genome, is considered a top-notch candidate for biomass production due to its salient features viz. fast growth rate and abilities for high tillering, ratooning, and photosynthesis. Energy cane, an ideal type of sugarcane, has been bred specifically as a biomass crop. In this review, we described (1) biomass potentials of sugarcane and its underlying genetics, (2) challenges associated with biomass improvement such as large and complex genome, narrow gene pool in existing commercial cultivars, long breeding cycle, and non-synchronous flowering, (3) available genetic resources such as germplasm resources, and genomic and cell wall-related databases that facilitate biomass improvement, and (4) mining candidate genes controlling biomass in genomic databases. We extensively reviewed databases for biomass-related genes and their usefulness in biofuel generation. This review provides valuable resources for sugarcane breeders, geneticists, and broad scientific communities involved in bioenergy production.

## Sugarcane, a Potential Biofuel Crop

Sugarcane (*Saccharum* spp.) is a perennial, tropical or subtropical non-cereal grass mainly grown for sugar, contributing to approximately 75% to total global sugar production (Commodity Research Bureau, 2015). It has a close genetic relationship with sorghum (*Sorghum bicolor*) and other members of *Poaceae* family, namely, *Miscanthus* (*Miscanthus x giganteus*) and *Erianthus* (*Erianthus arundinaceus*) (Amalraj and Balasundaram, 2006). As a C4 plant, sugarcane has one of the highest solar energy conversion efficiency and highest biomass yield among the known crops (Henry, 2010; Byrt et al., 2011). Biomass accumulation in sugarcane could reach up to 550 kg/ha/day (Muchow et al., 1994). More recently, sugarcane has been increasingly exploited as a second-generation (i.e., lignocellulosic-based) biofuel feedstock (Lam et al., 2009). Sugarcane had the highest dry biomass yield (39 t/ha/yr), followed by *Miscanthus* (29.6 t/ha/yr*)*, maize (17.6 t/ha/yr), and switchgrass (10.4 t/ha/yr) (Heaton et al., 2008), which could vary depending on the growing season and conditions. The average dry lignocellulosic biomass yield of sugarcane was approximately 22.9 ton/ha/yr (van Der Weijde et al., 2013) with some exceptional genotypes reaching up to 80–85 ton/ha/yr (Moore et al., 1998) and theoretical potential yield can exceed 100 ton/ha/yr (Jakob et al., 2009; Moore, 2009). Sugarcane has been grown in more than 100 countries in the world with Brazil, India, and China as the top sugarcane producers (FAOSTAT, 2016).

### Sugarcane Biomass Potentials

Biomass, an alternative source to fossil resources, offers a promising opportunity for renewable energy (Lynd et al., 2008). Plant biomass, specifically lignocellulose, composed of plant cell walls from grass family, is considered sustainable and renewable feedstock for biofuels (Ragauskas et al., 2006). This concept prompted the establishment of biomass industries across the globe (**Table 1**). Sugarcane is a standout among the bioenergy crops for bioethanol production because of its fast growing and high biomass yielding capacity (Waclawovsky et al., 2010). Sugarcane biomass mainly comes from stalks and straw which respectively constituted 80–85% and 10–15% of total biomass (Carvalho-Netto et al., 2014). Tops, the plant parts between the upper end and the last stalk node with attached green leaves, constituted up to 26% of the total stem weight at harvest (Miocque, 1999).

**Table 1:** Biomass-related databases.

Database	Link	Country	Purpose
Biomass Energy Centre	http://www.biomassenergycentre.org.uk/portal/page?_pageid=73,1&_dad=portal&_schema=PORTAL	United Kingdom	Bioenergy
European Biomass Industry Association	http://www.eubia.org/	European Union	Bioenergy
Louisiana Biomass Resources Database	http://www2.lsuagcenter.com/biomass/about.aspx	United States	Bioenergy
Biomass Power Association	http://www.biomasspowerassociation.com/	United States	Electricity
Sugarcane	http://sugarcane.org/	Brazil	Bioenergy
SAHYOG Project	http://www.sahyog-europa-india.eu/	European Union and India	Bioenergy
BioEnergy Science Center	http://www.bioenergycenter.org/besc/	United States	Cellulosic biofuels
Russian Biofuel Association	http://www.biofuels.ru/	Russia	Bioethanol, biodiesel

The oil crisis in the US in the late 1970s spurred the use of sugarcane as an energy plant, (Alexander, 1985; Bischoff et al., 2008). Energy cane, an ideal type of sugarcane showing high biomass yield, was specifically selected for biofuel production (Knoll et al., 2013). Two very distinct traits of energy canes included high number of tillers or stalks per stool and vigorous ratooning ability (Matsuoka and Stolf, 2012). Compared to conventional sugarcane, energy cane hybrids produced 138 and 235% more total biomass (green matter) and fiber, respectively (Matsuoka et al., 2012). With the availability of technologies that convert lignocellulosic biomass into ethanol, the cultivation of energy cane is recently widely increasing (Carvalho-Netto et al., 2014). This emerging biofuel crop is currently being expanded commercially to achieve an annual yield target of one million tons of cane in Florida State alone.

Energy cane has been divided into Type I and Type II physiological types based on its sugar and fiber content (Tew and Cobill, 2008). Type I energy cane contains comparable level of sugar (>13%) but higher fiber content (>17%) than conventional sugarcane. In contrast, Type II energy cane has marginal sugar content (<5%) but very high fiber content (>30%) and is exclusively bred for biomass production. Lignin content in Type I and Type II energy canes was slightly more than that of conventional sugarcane (Knoll et al., 2013). Energy cane fulfills all the requirements for a renewable bioenergy source (Matsuoka et al., 2014). In marginal land of low-fertility where sugarcane cultivation is not profitable, growers may consider growing energy cane for lignocellulosic ethanol production (Sandhu and Gilbert, 2014). Recently, energy cane hybrids of both Type I and Type II varieties are being developed by various private breeding companies in Brazil (Matsuoka et al., 2014). These energy cane varieties can be expanded in geographical range beyond tropical and subtropical regions owing to its wider adaptation and cold tolerance characteristics (Knoll et al., 2013; van Antwerpen et al., 2013).

### Sugarcane Biomass Quality

The second-generation bioethanol production not only depends on cellulose content of biomass, the major component for biofuel production, but also on the quality of plant cell wall. Cellulose accounted for about 43–49% of above-ground dry matter in sugarcane and energy cane cultivars (Sanjuan et al., 2001; Kim and Day, 2011), which is comparable to wood (∼45%; Smook, 1992) and more than typical forage grasses (∼30%; Theander and Westerlund, 1993). Plant cell wall is composed of ‘complex and dynamic extracellular matrices’ that regulate cell growth, provide mechanical support, and protect against pathogens. There are two types of plant cell wall on the bases of the architecture, chemical composition, and biosynthetic processes involved (Carpita, 1996). Primary cell wall is formed by deposition of polysaccharides, predominantly cellulose, hemicellulose, and pectin (Cosgrove, 2005). Secondary cell wall (SCW) is deposited inside primary wall to provide mechanical strength after cells cease to grow and accounts for most of the biomass for biofuel production.

The SCW in sugarcane is composed of mostly cellulose (∼50%), lignin (∼25%), and hemicellulose (∼25%) (Loureiro et al., 2011). Cellulose and hemicellulose serve as the skeletons of plants and are further ‘strengthened by lignin and phenolic cross-linkages’ (Carpita, 1996). Cellulose and hemicellulose are composed of different carbohydrate polymers and can be converted into fermentable sugars for bioethanol. However, this requires chemical processes such as pretreatment and enzymatic hydrolysis of cellulose, depolymerization, and distillation. Lignocellulosic biomass is recalcitrant to bioethanol conversion, mainly due to lignin and monolignol in cell wall (Weng et al., 2008; Vanholme et al., 2010). Lignification process in sugarcane when studied using histological, biochemical, and transcriptional data obtained from two sugarcane genotypes with contrasting lignin contents, revealed a total of 35 compounds that were related to lignin biosynthesis in sugarcane stems (Bottcher et al., 2013).

Besides composition and content of lignin, composition, structure, and interactions of other polysaccharides in the cell wall play a vital role in the efficient conversion of lignocellulosic biomass to ethanol. Various studies have reported that ‘degree of cell wall porosity,’ ‘cellulose crystallinity,’ ‘polysaccharide accessible surface area,’ and ‘protective sheathing of cellulose by hemicellulose’ contributed to recalcitrance of cell wall to ‘enzymatic degradation’ (Himmel et al., 2007; Gross and Chu, 2010; Zhang et al., 2012; Zhao et al., 2012). Understanding the biochemistry of cell wall, genes involved in its biosynthesis, and development of sugarcane genotypes to fulfill the requirements for efficient conversion of biomass to ethanol should be the focus of sugarcane bioethanol production in the future.

### Genetic Studies of Sugarcane Biomass

Biomass yield in sugarcane could be improved by an enhanced understanding of underlying genetics of biomass yield components: stalk number (SN), stalk diameter (SD), stalk height (SH), and stalk weight (SW). These components were controlled by genes with additive and non-additive effects and their interactions (Zhou et al., 2009; Carvalho-Netto et al., 2014). Relative contribution of additive variance was the highest for SN among these components. Similarly, high genetic variability and heritability existed in sugarcane for SD, SN, and SW (Sanghera et al., 2014), implying that selection of sugarcane clones for biomass trait is feasible. Further, an attempt was made to identify the quantitative trait loci (QTLs) controlling biomass yield components such as SH, SN, SD, and brix with a population consisting of 295 progeny developed by selfing ‘R570’ (Hoarau et al., 2002). A total of 40 putative quantitative trait alleles (QTAs) were identified, with each QTA contributing only 3–7% toward total phenotypic variation. Another effort made by Aitken et al. (2004) reported 32 putative QTLs associated with SN, SD, and SW in an F_1_ segregating population. Similarly, the phenotypic variations explained by each QTL were very low, ranging from 3 to 9%. Interestingly, 11 of the 32 QTLs identified were associated with more than one trait. Molecular markers linked to biomass yield components have been identified and thus could be used in introgression breeding programs. Recently, an association mapping conducted on 28 genotypes of sugarcane identified a few simple sequence repeat (SSR) markers associated to SW and SN (Bilal et al., 2015). So, numerous biomass yield components of sugarcane can be targeted to enhance biomass production. Recently, gene expression analysis showed that 1,649 and 555 differently expressed (DE) transcripts were revealed between young and mature tissues and between 10 sugarcane genotypes with different level of fiber content, respectively. Of these DE transcripts, 151 and 23, respectively were directly related to fiber and sugar accumulation. In addition, the analysis also found full-length candidate transcripts and pathways that could determine the contrasting fiber accumulation in genotypes with varying content and tissue types (Hoang et al., 2017). The results from gene expression analysis is more reliable than that of molecular marker analysis as it offers the ability to discriminate closely related gene transcripts (Hoang et al., 2017). Thus, biomass yield improvement in sugarcane could be feasible if we could couple the information on molecular markers linked to QTLs controlling biomass yield components with gene expression analysis.

The high tillering ability usually corresponded to an increased number of harvestable stalks and consequent production of high number of favorable ratoons in the following seasons (Matsuoka and Stolf, 2012). Thus, tillering has been considered as a critical biomass trait. Dissecting the genetics of tillering ability based on the information available in other species can also aid the effort in utilization of various genetics approaches for biomass improvement of sugarcane. Four QTLs that control tillering in sorghum were identified (Hart et al., 2001). Importantly, markers associated with SN in sugarcane have been identified, which were ‘co-located within or near QTLs that control tillering and rhizomatousness in sorghum’ (Jordan et al., 2004). Tillering characteristics in maize was reported to have an incomplete dominance (Rogers, 1950). Two genes g*rassy tiller1 (gt1)* and *teosinte branched1 (tb1)* acted in a common pathway that control tillering in maize (Whipple et al., 2011). A homolog of *tb1* gene in sorghum (Kebrom et al., 2006), *BRANCHED1* (*BRC1*) in *Arabidopsis* controlled formation of axillary buds. Similarly, *MONOCULM 1 (MOC1)*, likely a ‘master regulator’ of tillering has been isolated and characterized in rice (Li et al., 2003). Over-expression of *tb1* gene in rice reduced SN, though formation of axillary buds was not affected (Takeda et al., 2003). Targeted mutagenesis to *tb1* gene using CRISPR/Cas9 in switchgrass resulted in mutant plants with increased tiller production compared to wild types (Liu et al., 2017). With the homology and gene function conservation across grass species, most likely *tb1* gene would control tillering in sugarcane. Pribil et al. (2007) reported that plants with over-expressed sugarcane *tb1* were significantly taller than untransformed lines. However, SN was not significantly different between transformed and non-transformed lines. An effect of manipulating gibberellins (GA) metabolic pathway in the shoot architecture of sugarcane was also studied by Pribil et al. (2007). The genetically transformed sugarcane lines with over-expression of *GA 2-oxidase*, coding an enzyme that converts GA into non-functional GA, in the cultivar Q117 exhibited variations in height reduction (47 ± 4 cm) and tiller production (5 ± 0.6) relative to control plants (174 ± 21 cm; 1.8 ± 0.9). In contrary, over-expression of *GA 20-oxidase* gene increased stem elongation and stem weight, while substantially reducing SN. In another effort by Pribil et al. (2007), the data obtained from a total of 31 Q117 transgenic sugarcane lines produced with reduced expression of another branching gene, *MAX3*, involved in strigolactone biosynthesis, indicated that regulation of axillary branching affect plant height in sugarcane. These studies suggested that tillering characteristics in sugarcane could be manipulated by introgressing the genes that control tillering. However, with the complex genomes in sugarcane and species-specific genetic composition, the gene interaction network, dosage effects, and various genetic backgrounds of the recipient clones could remarkably complicate the gene effects after introgression and manipulating process in sugarcane.

## Challenges of Sugarcane Biomass Improvement

Biomass yield is a complex concept. Broadly speaking, goals of sugarcane breeders should be to enhance overall biomass yield, biomass quality, and adaptation to wider environment etc. The biomass yield trait could be explained at three levels, and is usually intertwined with biomass quality. At the field level, the biomass trait is dry biomass yield per acre, which is determined by planting density if plant genotype is fixed. At the individual plant level, biomass can be further dissected into SH, SD, SN, and leaf biomass. Thus, selecting genotypes with enhanced SH, SD, SN, and leaf biomass is crucial for higher biomass yield. At the cellular level, cellulose, hemicellulose, and lignin in the cell wall constitute the plant biomass. Increasing the relative cellulose and hemicellulose content as well as balancing the lignin content was vital for increasing biomass yield and enhancing biofuel conversion efficiency (Li et al., 2014). Jung et al. (2013) reported a compromised biomass yield in sugarcane when *caffeic acid O-methyltransferase (COMT)*, a key enzyme in lignin biosynthesis, was suppressed by 91% and lignin content was reduced by 12%. However, 80% suppression of *COMT* and 6% reduction in lignin content made no impact on biomass yield significantly. So, integrating all these different levels of traits in one systematic crop is very challenging because it depends on identifying the genetic basis or components of each specific trait, and balancing those components.

Sugarcane biomass improvement faces additional and specific inherent challenges viz. narrow gene pool in modern sugarcane cultivars, poor synchronization and fertility of flowers in parental clones, long breeding/selection cycle, and genomic complexity (Manickavasagam et al., 2004; Lakshmanan et al., 2005). These issues have been hindering the ability of breeders to efficiently improve biomass traits and thus must be dealt with selection of parents with wide genetic variability, synchronous flowering and cross-fertility, coupled with molecular markers to improve the efficiency of genotype selection.

### Narrow Genetic Bases of Current Cultivars

Modern sugarcane cultivars were derived from only a handful of sugarcane clones (Arceneaux, 1967; Roach, 1989) including eight *Saccharum officinarum*, two *S. spontaneum*, one natural hybrid of *S. spontaneum* and *S. officinarum*, and two *S. sinense*. In addition, commercial cultivars were further developed from intercrossing of hybrids and their subsequent backcrosses to *S. officinarum*, called nobilization. These hybrids were repeatedly used in developing modern sugarcane cultivars, which contributed to narrow genetic bases of current sugarcane cultivars. Consequently, sugarcane cultivars became vulnerable to various diseases and insect pests in addition to a diminished genetic gain for both sugar content and biomass yield.

### Poor Synchronization and Fertility of Flowers

The synchronization in flowering between clones selected for crossing is very critical in sugarcane breeding programs. Sugarcane clones tend to flower up to 8 weeks apart (Nuss, 1982), and it is especially pronounced between *S. officinarum* and *S. spontaneum* (Moore and Nuss, 1987), thus requiring breeders to artificially induce flowering in an attempt to facilitate cross pollination. This lack of overlap in flowering periods between desired clones could debilitate the breeding programs. Thus, studies have been conducted to synchronize flowering in desired parents through manipulation of the photoperiod (Bull and Glasziou, 1979). In addition, sugarcane flowers have ‘low male fertility’ and reduced pollen viability at high latitudes (Moore and Nuss, 1987), and in some cases, were self-sterile (Skinner, 1959). Moreover, progeny derived from crosses involving high degree of self-pollination showed decreased viability and vigor (Skinner, 1959; Tew and Pan, 2010). Thus, selection of desired parents that are cross-fertile, yet with wide genetic distance to ensure fertile progeny with broader genetic base is critical in sugarcane improvement.

### Long Breeding/Selection Cycle

Hybridization in sugarcane is tedious, time consuming, and requires special skills to perform. Conventional sugarcane breeding takes 10–15 year to create new cultivars because sugarcane has a long growing season of 10–12 months (one generation/year). Basically, sugarcane breeding program involves three basic steps: (i) parental clone selection, (ii) hybridization, and (iii) selection of superior progeny in several vegetatively propagated generations based on their phenotypic performance (11 year). At early generations, selection of superior genotypes is performed for the traits with high heritability, albeit, using low selection intensity. Broad-sense heritability for biomass yield components such as SD, SN, and SH (Sanghera et al., 2014) and overall cane yield was high (0.51–0.84) (Racedo et al., 2016) in sugarcane and thus can be selected for in early generations. At later generation of selection, significantly reduced number of clones will be planted in replications at different environments for performance and thus helps increase the experimental accuracy to screen the traits with low heritability (Gazaffi et al., 2014). Final characterization involves further evaluation of selected genotypes for stability, uniformity, yield, and uniqueness by assessing over several cuts. Superior genotypes are then released as cultivars for commercial production.

### Genomic Complexity and Genome Size

Most commercial sugarcane cultivars are interspecific hybrids that have chromosome from 100 to130, with approximately 80% of chromosomes inherited from *S. officinarum*, 10–20% from *S. spontaneum*, and less than 5–17% from recombination between the two species (D’Hont et al., 1996; Piperidis and D’Hont, 2001; Cuadrado et al., 2004). Thus, each locus of sugarcane cultivars has up to 12 alleles (Le Cunff et al., 2008). The somatic cell genome (2C) size of the modern cultivar ‘R570’ (2n = 115) was approximately 10,000 Mbp (10 Gbp) (D’Hont, 2005) with an average size of 87 Mbp per chromosome, which is larger than the 73 Mbp per chromosome in sorghum (Wang et al., 2010). The monoploid sugarcane genome (750–930 Mbp) is twice the size of rice (389 Mbp), similar to sorghum (760 Mbp), and much smaller than maize (2500 Mbp) (D’Hont and Glaszmann, 2001). Thus, high polyploidy and large genome size pose considerable challenges in sugarcane improvement through QTL identification and marker assisted selection (MAS) (Sreenivasan et al., 1987; Lu et al., 1994; Jannoo et al., 1999).

Allele segregation and inheritance in sugarcane are much more complicated than diploid species. Multiple homologous chromosomes with multi-dose alleles commonly occur in *Saccharum* spp., which complicate the segregation ratio in the crosses and thus required evaluation of thousands of progeny to sort out the segregation of alleles (Matsuoka et al., 2009). In addition, large and complex genome required a large number of molecular markers to sufficiently cover the genome (Gouy et al., 2013). Consequently, development of markers linked with desirable traits is challenging tasks in sugarcane. Furthermore, selection of superior F_1_ hybrids with favorable alleles became difficult due to a substantial random sorting of homologous and homoelogous chromosomes and the formation of recombinants (Grivet and Arruda, 2002). Thus, genomic complexity hinders the dissection of biomass traits at the molecular level, complicating sugarcane improvement program through MAS. The current selection of sugarcane genotypes with improved biomass yield mainly relied on visual and labor-intensive field traits measurements.

## Genetic Resources for Sugarcane Biomass Improvement

### Sugarcane Germplasm and Their Utilization

Sugarcane germplasm collection is the potential source of genetic variation for many traits including biomass. For example, *S. spontaneum* possessed wide genetic variability for morphology, ratooning, and tolerance for biotic and abiotic stresses (Aitken and McNeil, 2010; Govindaraj et al., 2014). Modern sugarcane cultivars inherited the tillering and ratooning ability from *S. spontaneum* through hybridization (Matsuoka and Stolf, 2012). In addition, *S. spontaneum* is genetically more diverse than *S. officinarum*, thus contributing to ecological adaptation of sugarcane (Jackson, 1994; Tew and Cobill, 2008), which allowed sugarcane to grow even in marginal land. Sugarcane or energy cane breeding should tap into all the relevant information on genetic variances in the germplasm associated with biomass yield traits to not only improve but also to broaden the genetic base of biomass traits (Todd et al., 2014).

Organized attempts were made to collect genotypes that were highly productive, resistant to diseases, and had high sugar content (Berding and Roach, 1987). International Board of Plant Genetic Resources (IBPGR) and International Society of Sugar Cane Technologists (ISSCT) undertook efforts to collect sugarcane accessions (Anonymous, 1982) and consequently, two duplicated world sugarcane (*Saccharum* spp.) collections are maintained in India and USDA, known collectively as the ‘World Collection of Sugarcane and Related Grasses’ (WCSRG). The National Plant Repository in Miami, FL, United States maintains over 1000 accessions of *Saccharum* germplasm collected from 45 different countries all over the world (Berding and Roach, 1987; Comstock et al., 1995). The WCSRG contains enormous genetic variability for various morphological traits, biomass yield components, adaption to stresses, and other agronomic or quality traits. The WCSRG provides a repository for many valuable alleles of lignocellulosic biomass traits, which could be targeted to enhance biomass production through energy cane breeding directly or can be utilized for identifying alleles associated with biomass traits for marker development and MAS.

Characterization of germplasm serves as an important bridge linking the collection and utilization phases of germplasm conservation (Heinz, 1987). In efforts to use the WCSRG in breeding program and to broaden the genetic base of sugarcane cultivars, the genetic diversity analysis on partial genotypes in WCSRG was conducted (Tai and Miller, 2002; Brown et al., 2007). The CP 96–1252 was released with a widened germplasm base through introgression program among WCSRG (Miller et al., 2005). In addition, 1002 accessions from WCSRG, presumed to possess valuable alleles for biomass and other agronomic traits (Nayak et al., 2014), were genotyped with SSR markers. A core collection of 300 accessions that represented the genetic diversity of WCSRG was developed according to genotypic data (Nayak et al., 2014). On the other hand, the WCSRG was phenotypically characterized by eight traits and a similar core collection was developed based on morphological traits (Todd et al., 2014). A diversity panel representing the WCSRG were selected by weighing in different parameters from combination of both phenotypic and genotypic data, which, not only serves as an association population to discover the desirable alleles in the future, but also can be utilized in the breeding program for crop improvement as they have been thoroughly evaluated for various traits (Todd et al., 2017).

### Sugarcane Genomic Databases

Though sugarcane has a complex genome to decipher, sugarcane geneticists have invested significant efforts to explore and dissect its complex genome using different genomic tools. Genomic databases are critical reservoirs and important foundations for molecular breeders to mine the candidate genes and to facilitate molecular crop improvement through MAS. Below, we summarized the publicly available genomic databases (**Table 2**), which can be mined and utilized for sugarcane molecular improvement.

**Table 2:** Publicly available genomic resources and tools for sugarcane and its allied species.

Database	Link	Species	Type
Sugarcane transcription factor database	http://planttfdb.cbi.pku.edu.cn/index.php?sp=Sof	*S. officinarum*	Transcription factor
SUCEST-FUN	http://sucest-fun.org	Sugarcane	EST
TropGENE	http://tropgenedb.cirad.fr/tropgene/JSP/index.jsp	Tropical crops (banana, cocoa, breadfruit, coconut, coffee, cotton, oil palm, rice, rubber tree, sugarcane)	QTLs, genetic and physical maps, Phenotypes, Parentage, allelic diversity
Grassius	http://grassius.org/	*Brachypodium* maize, sugarcane, sorghum, and rice,	Transcription factor
Phytozome	https://phytozome.jgi.doe.gov/pz/portal.html	Eighty-six green plants	Whole genome sequences and annotation
Sorghum transcription factor database	http://planttfdb_v1.cbi.pku.edu.cn:9010/web/index.php?sp=sb	Sorghum	Transcription factor
MOROKOSHI	http://sorghum.riken.jp/morokoshi/Home.html	Sorghum	Transcriptome, FL-cDNA

SUCEST-FUN Database^1^ is a large sugarcane functional genomics database including approximately 237,954 expressed sequence tags (ESTs) from 26 diverse cDNA libraries constructed from different sugarcane varieties with different developmental stages and different tissues and organs (Vettore et al., 2003). The ESTs were further assembled into 42,982 distinct contigs, which had 71 and 82% of contigs significantly matching the *Arabidopsis* and rice genome, respectively. The database webserver integrates transcripts, molecular markers, gene categories, gene expression studies, and data mining tools to provide comprehensive access to sugarcane genomic resources (Nishiyama et al., 2012). This is the most comprehensive web portal for sugarcane genomic resources as it houses not only the sugarcane transcript sequences but also other related databases such as Sugarcane Gene Index (SGI), and Sugarcane Signal Transduction (SUCAST), and Sugarcane Metabolism (SUCAMET) as well.

Sugarcane transcription factor database^2^ has a collection of 1,177 predicted sugarcanes (*S. officinarum*) transcription factors (TFs). It is a part of plant transcription factor database (plantTFDB)^3^, which in turn catalogs all the genes involved in plant transcriptional activities and provides a repository for 320,370 putative TFs from 165 species (Jin et al., 2017) including sorghum, a close diploid relative of sugarcane, detailing ontology, domain feature, expression pattern, and orthologous groups of genes (Zhang et al., 2011). This database sheds light on interactions between TFs and target genes in order to explore functional mechanisms of TFs.

GRASSIUS^4^ is a publicly available web resource that integrated different databases as well as computational and experimental resources related to the control of gene expression in the grasses and associated agronomic traits and also links four databases: GrassTFDB (Grass transcription factor database), GrassCoRegDB (co-regulator database), GrassPROMDB (promoter database), and TFome collection (TF open reading frame) as well. GRASSIUS provides information on TFs from maize, sugarcane, rice, sorghum, and *Brachypodium distachyon* and contains the collection of grass TFome, which provides information on full-length ORFs. GrassPROMDB furnishes the data on promoters and cis-regulatory elements for the aforementioned grass species (Yilmaz et al., 2009). Overall, it contains 9,044 TFs, 579 co-regulators, 149,075 promoter sequences, 2,114 TF ORF clones and 180 TFomes from five grass species. Recently, TFome for maize has been updated with 2,017 unique maize TFs including 24 families of co-regulators (Burdo et al., 2014). So, GRASSIUS especially focuses on regulatory elements and their interactions in grass species and can be utilized as backup sources and cross species comparative genome studies in sugarcane.

TropGENE^5^ database is a genetic information system for tropical crops. The most commonly stored information on this database included the genetic resources (agro-morphological traits, parentages, reactions to diseases and drought, and allelic diversity), molecular markers, genetic maps, sequences, genes, QTLs information, physical maps, and corresponding references (Ruiz et al., 2004; Hamelin et al., 2013). It contained about 19,800 molecular markers and 9,500 germplasm entries for 10 tropical crops with their accession number, country of origin, taxonomy, ploidy level, and phenotypic information on agronomic and morphological traits (Hamelin et al., 2013). TropGENE differs from other sugarcane-related databases in that it provides both genetic and phenotypic resources for tropical crops including sugarcane. Thus, a typical agronomic trait can be explored at both molecular and phenotypical levels in this database.

Phytozome^6^ serves as a comparative portal for green plant genomics. It is a centralized platform that provides evolutionary history of plant gene at the sequence level in addition to offering information on gene structure, gene family, genome organization, and functional annotations of complete plant genomes (Goodstein et al., 2012). Sorghum belongs to the same subtribe Saccharine as sugarcane which makes a reliable model because of its small genome (730 Mbp) for functional genomics of sugarcane and other C4 grasses. Besides, its high level of inbreeding and the partitioning of carbon into sugar make it a model for biomass crops like sugarcane (Paterson et al., 2009). About 85% of sorghum genes are orthologous to sugarcane genes thus sorghum genome provides an excellent resource to study sugarcane genome (Wang et al., 2010). Currently, of the 86 sequenced and annotated plant genomes, 52 have been clustered into gene families at 15 evolutionarily significant nodes^6^. In addition to comparative genomics, phytozome also provides information on expression data and proteome of different organisms. It is the most comprehensive database for retrieving green plant genomes.

### Cell Wall Composition Databases of Related Species

Because lignocellulose is very recalcitrant to enzymatic degradation, bioenergy researchers should have the knowledge of the genes particularly involved in its biosynthetic pathways so that those genes could be selected or modified to achieve readily degradable biomass (Ekstrom et al., 2014). In quest for efficient conversion of lignocellulose into ethanol, many cell wall-related databases have been developed and updated regularly with new findings on cell wall genomics. These databases will be excellent resources for comparative genomics study in identifying target genes (Saballos, 2013) for biological and genetic studies and for biofuel crop improvement (Yin, 2014). The plant cell wall-related databases^7^ were divided into general, species-specific, and family specific databases (reviewed by Cao et al., 2010). We provide brief discussions on these databases as in-depth review for most of the databases is provided previously (Cao et al., 2010).

#### General Databases Provide Information about Cell Wall-Related Genes and Their Biosynthetic Pathway for Different Species

Cell wall genomics (CWG)^8^ was created and maintained by collaborative efforts of scientists at different universities and research institutions. CWG is supported by the NSF Plant Genome Research Program and provides huge resources for plant biologists studying mutants of ‘cell wall-related genes’ in *Arabidopsis*, rice, maize, and sorghum. Specifically, this database provides the information on cell wall biogenesis pathway, T-DNA insertional mutants, and forward and reverse genetics for insertional mutants. CWG characterizes the cell wall phenotypes of homozygous cell wall mutants of *Arabidopsis* (dicot) and maize (monocot), providing large scale insertional DNA lines for both plant species as well as characterizing the genes associated with architectural assembly of the cell wall. Despite the lack of functional annotation, an estimated 1,000 genes were reported to be involved in biosynthesis of cell wall-related proteins (Yong et al., 2005). CWG provides information on gene families involved in cell wall biogenesis for both monocot (maize) and dicot (*Arabidopsis*) plant species. Six stages of cell wall formation have been outlined including substrate generation, synthases and glycosyl transferases, secretory pathway, wall assembly, wall dynamics, and wall disassembly. Basically, CWG is a complete repository for gene families and their pathways involved in cell wall formation.

Cell wall navigator (CWN) integrates cell wall-related protein families from many plant and non-plant species, allowing comparison of sequences derived from different species. It has four unique features; (1) an adaptive design for organizing complex protein families from many organisms to cover all the known sequences, (2) a flexible architecture to integrate new families rapidly, (3) an automated update and analysis pipeline for maintaining current information, and (4) many visualization and interactive mining tools. It has information for more than 30 gene families comprising more than 5,000 coding genes involved in primary cell wall metabolism. It incorporates sequences from three different resources: *Arabidopsis* and *Oryza sativa* sp. *japonica* from The Institute for Genomic Research (TIGR), the UniProt database, and the EST division of the National Center for Biotechnology Information (NCBI). The organism-unspecific EST search tool allows the comparative genomic study of novel genes in organisms with distinctive cell wall compositions (Girke et al., 2004). Thus, CWN provides information on detailed functional genomic data involved cell wall biosynthesis as opposed to CWG.

Plant cell walls^9^ was created and maintained by complex carbohydrate research center (CCRC) at the University of Georgia (UGA). The CCRC in turn was founded in 1985 at UGA to better understand the chemical structure and biological functions of complex carbohydrates. The research was carried out by six independently funded groups that studied various areas including primary cell wall structures, three-dimensional conformations of cell wall components, the interactions and biosynthesis of cell wall components, and functional role of cell wall as a barrier to plant pathogens and source of biofuels. Plant cell walls focuses on cell wall formation with regard to structural, mechanical, and defensive roles mostly at the biochemical level.

Plant database of annotated cell wall genomes contains genome information on annotated genes, gene structures, and protein functions for seven plant genomes (e.g., rice, *Arabidopsis*, sorghum etc.), 12 algal genomes, as well as individual proteins encoded in these genomes. The information on cell wall-related gene families such as carbohydrate active enzyme (CAZy) family, protein family (Pfam) domain information, 3-D protein structures, homology-based functional prediction, phylogenetic trees of CAZy family proteins (133 CAZy), and their subcellular localizations and interactions allows users to conduct comparative genomic analyses of cell wall-related genes (Mao et al., 2009). This database analyzes only annotated cell wall-related genes for comparative genomics.

CAZy database^10^ is the most comprehensive repository of Carbohydrate-Active enZYmes (CAZymes) (Park et al., 2010), an important class of proteins that synthesizes, modifies, and degrades structural and storage biomass polysaccharides (Cantarel et al., 2009). Thus, knowledge of CAZymes is crucial to biofuel industry (Yin et al., 2012). The database comprised five classes of protein families: glycosyltransferases (GTs), polysaccharide lyases (PLs), carbohydrate esterases (CEs), carbohydrate-binding modules (CBMs), and glycoside hydrolases (GHs). CAZy provides genomic, biochemical, taxonomical, and structural information on many cell wall-related proteins while providing sequence annotation information from other publicly available resources. It contains the regularly updated information on CAZy protein family, incorporation of new family members and their biochemical information obtained from literature. It reports sequence information for about 340,000 CAZymes, which includes 12,700 biochemically characterized CAZymes and 1400 CAZymes with 3D structures (Lombard et al., 2014). Further, CAZymes Annotation Tools (CAT) was developed for systematic annotation of CAZy proteins. CAT utilizes information collected in the CAZy database, analyzes it, and supplements it with information from other databases (Park et al., 2010). As of November 2017, the database contains CAZymes information for 8,436 Bacteria, 283 Archaea, 212 Eukaryota, and 332 Viruses. Basically, CAZymes studies storage biomass polysaccharides that are directly involved in plant biomass formation.

Database for automated carbohydrate active enzyme annotation (dbCAN^11^) is an improvement on CAZy database in a way that it provides an automated and comprehensive annotation of CAZymes in addition to an easy access to sequences, signature domains, alignments, and phylogeny data of CAZyme-related enzyme families (Yin et al., 2012). PlantCAZyme^12^ is a web resource built upon dbCAN and is especially dedicated to providing pre-computed sequence and annotation data on CAZymes. It has information on 43,7900 CAZymes of 159 protein families from 35 plants and chlorophyte algae of fully sequenced genomes (Ekstrom et al., 2014).

#### Species-Specific Databases

Species-specific databases provide information on cell wall-related genes for particular species. Thus, they complement the general database for deeper understanding of cell wall genes for the species (Cao et al., 2010).

MAIZEWALL^13^ provides a public repository on ‘a bioinformatic analysis and gene expression data’ related to ‘cell wall biosynthesis and assembly in maize.’ It has 735 contigs that have been classified into 174 gene families and which in turn are classified into 19 functional cell wall-related categories based on known gene annotations. Of the 735 contigs, 651 have full set of developmental gene expression data. Gene expression data are easily accessible and are ranked based on their expression level for each organ and internode stage. Maize homologs were obtained based on 100 cell-wall related keywords and BLAST search against the available cell wall-related genes and homology search against ESTs obtained from cell wall-forming TEs in Zinnia (Guillaumie et al., 2007). MAIZEWALL ‘allowed alignments of multiple sequence, identification of predicted protein domains, and sub-cellular localizations of target sequences using user-friendly bioinformatics software’ (Cao et al., 2010). In addition, it provided the complete bioinformatic information of each gene as well as gene-specific tags and organ specific fingerprint of each cell wall-related gene (Guillaumie et al., 2007).

Wheat GlycosylTransferase Inventory database (GTIdb^14^) has been used for searching exhaustive candidate genes in wheat that play roles in particular biological process. It provides comprehensive analysis of glycosyltransferases (GT), a multi-gene superfamily involved in biosynthesis of cell wall and storage polysaccharides plus glycosylation of various metabolites. Wheat GT sequences were identified based on sequence homology with *Arabidopsis* and rice GT’s found in CAZy database. The database is comprised of two sections: the wheat section and the core database. A total of 912,573 wheat ESTs extracted from 220 EST libraries were used to ‘characterize 833 contigs and 2,296 singletons into 41 GT families.’ The database provides the sequences of GT for wheat, *Arabidopsis*, and rice in a downloadable format. In addition, phylogenetic trees that provide information on each family of GT from all three species are available in PDF format (Sado et al., 2009).

Rice GT database^15^ integrates and hosts functional genomic data for putative rice GTs. It displays user-selected functional genomic data on phylogenetic tree that included sequence and mutant lines information, and expression data. In addition, interactive chromosomal map delineating positions of GTs are included. There are 617 putative GT genes that corresponded to 793 transcripts (gene models) in rice. Links are provided to BLAST, CAZy database, Rice Annotation Project Database (RAP-DB), MSU/TIGR rice database, GRAMENE database, and other rice related databases. Of the 33 rice-diverged GT genes that expressed strongly in above-ground, vegetative tissues, 21 were strong candidates for understanding and manipulating cell walls for biofuel production (Cao et al., 2008).

#### Cell Wall-Related Gene Family Databases

Expansin Central^16^ provides information solely on expansin proteins. Expansins, involved in cell growth, cell wall disassembly, cell separation, and cell wall loosening, are plant cell wall proteins (Cho and Kende, 1997a,b; Li et al., 2003). Expansin central details on protein structure, mechanism of action, nomenclature, genes involved in biosynthetic pathway, protocols, phylogenetic tree, and references for expansin genes. Currently, the database contains a total of 226 expansin gene sequences for *Arabidopsis*, rice, maize, tomato, papaya, poplar and many other species.

Xyloglucan endotransglycosylases/hydrolases (XTH World) provides wealth of information related to composition and organization of primary cell wall and its spatial and temporal variability. In addition, it gives the information on how different cell wall microfibrils interact to form the primary cell wall in dicotyledonous plants as well as different genes involved in cell wall biosynthesis in rice, *Arabidopsis*, tomato, and other crops. To avoid the confusion due to contradictory series of nomenclature for essentially the same class of genes or proteins, the unifying nomenclature was proposed to classify a class of genes that encoded a spectrum of biochemical activities under xyloglucan endotransglucosylase/hydrolase (Rose et al., 2002). Xyloglucan binds cellulose non-covalently and also cross-links cellulose microfibrils (McCann et al., 1992). The database focused on standardized nomenclature and systematic identification of genes/proteins that fell under *xyloglucan endotransglucosylase-hydrolases* (*XTH*) gene family (Cosgrove, 2005). In addition, it provides the links to different databases for rice, tomato, and *Arabidopsis*.

Glycoside Hydrolases Database (GHDB) provides information on CAZy family GH16 glycoside hydrolases, including sequences of 260 amino acids that belong to the family. It provides 3D protein structures, functional annotation, phylogenetic trees, multiple sequence alignments of subfamilies: GH16a and GH16b, and homologous subgroups (Strohmeier et al., 2004). In addition, automatic BLAST search was also incorporated into the database in order to provide comprehensive analysis of the stored data (Strohmeier et al., 2004).

In summary, CWG and CWN databases have exclusive information on cell wall biogenesis pathways in general and are easily accessible. CWN provides the comparative study on sequences of protein families from different plant species that are involved in plant cell wall metabolism. ‘Plant cell walls’ is good resource for the scientific community interested in biofuel potential of cell wall whereas ‘plant database of annotated cell wall genomes’ is a huge resource for comparative genomic study of cell wall-related genes across plant and non-plant genomes. CAZy database is a resource dedicated to CAZy protein family involved in cell wall synthesis across all kingdoms, such as Bacteria, Archea, Eukayota, and Viruses. It is the most useful cell wall database for bioenergy research as CAZymes are the integral parts of cell wall biosynthesis. The dbCAN along with PlantCAZyme and CAZy database are dedicated to providing information on CAZymes to enhance bioenergy related studies. ‘MAIZEWALL’ solely delves into the biosynthesis of maize cell wall through transcriptome analysis of different developmental stages of maize. Wheat GTIdb focuses on candidate genes in wheat that play a key role in cell wall formation and storage polysaccharides. Rice GTdb is dedicated in integrating and hosting functional genomic data on GT genes, candidate genes for biofuel traits in rice. Expansin Central mainly focuses on expansin protein. The XTH database provides information primarily on XTH compound and its role in architectural assembly of the primary cell wall. The GHDB is a database that provides functional annotation and multiple sequence alignments of glycoside hydrolase enzymes of CAZy family.

### Application of Genomic Databases for Sugarcane Biomass Improvement

In the past decade, sugarcane became an attractive feedstock for second-generation biofuel production. Due to its complex genome structure and genetic inheritance, the genome sequencing progress is slow. In this vein, public genomic databases of related species and database searching tools provide powerful queries to get insight into biomass related genes from *Saccharum* genome before its whole genome sequence information is released.

#### Search for Biomass-Related Candidate Genes

Genetics and genomics of model species have uncovered many genes underlying the architecture of biomass yield components at individual plant level such as tillering pattern, SH, SN, leaf number and area, and structure and size of reproductive organs (Long et al., 2006; Jahn et al., 2011). Though we have summarized the sugarcane genomic databases and cell wall related databases, the plant architecture related database is currently not available yet. To retrieve plant architecture genes in sugarcane genome, the first step is to identify the candidate genes to form a candidate gene pool. Keywords defined based on relevant literature description of genes involved in plant architecture, such as tillering, vegetative growth, flowering time, leaf morphology, and secondary xylem and tracheary element differentiation can be used to search the published literature related to characterization of genes associated with biomass production. After evaluating the evidence presented in the paper, the gene sequences can be downloaded from the sources provided to form a plant architecture gene pool. Then the summarized sugarcane genomic databases can be searched through sequence blasting. The first databases to be searched can be the updated genomic sequences (CDS or protein sequences of the annotated gene models) of *Sorghum bicolor*, the closest species to sugarcane with a complete genome sequence from Phytozome database. The top hits are basically the corresponding nucleotide and protein sequences of the candidate genes in sorghum genome, which can then be BLASTed against the available sugarcane EST databases (**Table 2**) to retrieve the sugarcane nucleotide sequences.

Besides the genes involved in the plant architecture, genes related to cell wall biogenesis are important factors controlling biomass. Although many genes putatively involved in different aspects of cell wall biogenesis have been identified in a variety of model plants, relatively few genes contributing to biomass have been explicitly identified in *Sorghum bicolor*. Plant cell wall related gene databases can be searched. For example, the CWN, CWG, and MAIZEWALL databases classify cell wall-related genes into different categories: substrate generation, polymer synthesis, secretion, assembly, rearrangement during development, and disassembly (Girke et al., 2004; Penning et al., 2009). These databases give us an inventory of the genes that could become possible targets in the production of biomass. In order to obtain *Sorghum bicolor* homologs, *Arabidopsis* (6093), Rice (2002) and Maize (734) cell wall genes can be combined and used to BLAST search for their corresponding coding sequences in sorghum genome, then the transcript sequence in sugarcane through blasting the sugarcane related genomic databases (**Table 2**).

In sugarcane, a huge number of ESTs contain characterized candidate genes involved in important agronomic traits such as sucrose accumulation, biomass yield, and plant architecture etc. (Souza et al., 2001; Kido et al., 2012). Gene expression profiling database allows mining of large number of genes associated with biomass traits. For example, the sugar metabolism related genes have been assessed by transcriptome analysis to reveal the regulation of metabolic enzymes and sugar transporters in sugarcane stem (Casu et al., 2003, 2004, 2007; Watt et al., 2005). Cellulose synthase (*Ces*A) and cellulose synthase-like (*Cs*l) families were identified from 119 differentially expressed genes and further characterized in sugarcane (Casu et al., 2007). In two genotypes IACSP04-065 and IACSP04-627 with different lignin content, more than 2,000 transcripts along with genes that control lignin biosynthetic pathway showed differential expression, which can help us identify genes from the lignin biosynthesis and its interactions (Vicentini et al., 2015). The expression profile was analyzed between two genotypes contrasting for lignin content which showed that transcription factor ShMYB58/63 was correlated with ratio of Syringyl (S) and Guaiacyl (G) lignin substructures and interaction between ShMYB58/63 and ShF5H (Santos Brito et al., 2015). In addition, the EST database has proven to be a useful resource to discover sequence polymorphism in three genes of alcohol dehydrogenases (*Adh*) family (Grivet et al., 2003).

With the candidate gene pool, after exploring all the related databases, candidate gene association analysis can be conducted to identify alleles contributing to sugarcane biomass in a large sugarcane germplasm diversity panel with biomass traits and candidate gene sequence variations. Markers associated with biomass traits can be developed from the association analysis. MAS comes in handy especially to improve crops such as sugarcane that is propagated vegetatively and takes many years of selection for varietal development. QTLs for biomass traits can also be interrelated by the candidate genes in the QTL intervals. A substantial progress has been made to identify molecular markers linked to key biomass-related traits. Molecular markers linked to QTLs for biomass traits such as SD, SW, SN, and SH have been identified in prior studies (Hoarau et al., 2002; Aitken et al., 2004; Bilal et al., 2015). These molecular markers if validated could be utilized to select seedlings that possess QTLs controlling biomass yield traits. Selection of genotypes in seedling stage speeds up the breeding cycle and genetic gain. Besides, selection for these traits could be carried out in early generations because of their high heritability. An incorporation of desirable alleles from diverse germplasm into elite cultivars through MAS leads to improved genetic gain in the breeding programs. So, future studies should focus on molecular markers utilization, targeted mutagenesis, and gene expression analysis for introgression of genes that control biomass yield.

#### Modification of Biomass-Related Candidate Genes

Breeding endeavors in the future should focus not only on the high biomass yield of sugarcane, but also for high quality of biomass. Sugarcane biomass composition has been genetically modified to increase cellulose and hemicellulose content while balancing the lignin content for enhanced biofuel conversion efficiency (Li et al., 2014). *Cinnamyl alcohol dehydrogenase (CAD)* and *COMT* are two key enzymes involved in lignin synthesis. Plant growth and development were not affected when these enzymes were manipulated. However, doing so would change the quality and composition in cell wall (Saathoff et al., 2011). Additionally, transgenic sugarcanes produced increased sucrose and fiber contents in immature internodes, when activities of *pyrophosphate: fructose 6-phosphate 1-phosphotransferase (PFP)* were down-regulated (Groenewald and Botha, 2008; Van der Merwe et al., 2010). Transgenic sugarcanes with bacterial isomerase gene had a doubled sugar content, as well as ‘increased photosynthesis, sucrose transport and sink strength’ (Wu and Birch, 2007). Recently, engineering of lignin biosynthesis pathway genes by modulating lignin content has been a strategy to reduce the costs of enzymatic digestion of cellulosic biomass and improve cell wall digestibility. In fact, down-regulation of the *COMT* gene in sugarcane using RNA interference has shown decreased lignin content by 3.9–13.7% and thus required less enzyme and hydrolysis time to generate more fermentable sugar than control (Jung et al., 2012, 2013). Further, reduced cell wall lignin content improved enzymatic digestibility in sugarcane (Jung et al., 2012), maize (Park et al., 2012), and switchgrass (Fu et al., 2011; Saathoff et al., 2011; Yee et al., 2012). Though so much of the focus has been in down-regulating *COMT* or *CAD* genes in sugarcane, sorghum, maize, and switchgrass in order to reduce the lignin content, the improvement of sugarcane genotypes with improved lignocellulosic biomass quality is still at its infancy. As sugarcane has highly complex and polyploid genome, targeted mutagenesis using CRISPR/Cas9 could be a valuable tool to characterize target genes and sort out desirable genotypes. In addition, gene expression analysis could enhance the reliability of genes controlling biomass yield components.

## Conclusion

Sugarcane has a significant potential as a biomass crop due to its highly efficient photosynthetic rate, high tillering, and ratooning abilities. More recently, newly developed energy cane cultivars have higher fiber content and biomass yield than conventional sugarcane cultivars, specifically at marginal land, thus produce more second-generation biofuel.. However, most of the sugarcane and energy cane cultivars are hybrids developed from interspecific crosses of *S. spontaneum*, and *S. officinarum* with large and complex genome, which obscures molecular and genetic studies for crop improvement. In addition, narrow gene pool, non-synchronous and poor fertility of flowers among desired parents, and long breeding cycle bottleneck the efficient crop improvement for various economically important traits. Despite the challenges in sugarcane breeding, many genetic resources and genomic databases are available for the sugarcane biomass improvement at molecular level. Specifically, cell wall-related databases offer comprehensive information on biomass-related genes. Dissecting genes involved in biosynthesis of biomass polysaccharides help us better understand the biosynthetic pathways underlying primary and secondary cell wall synthesis, which will be helpful to improve the quality and yield of sugarcane biomass. The available genomic databases are valuable sources to aid studies for genetic improvement of sugarcane biomass quality and yield as the genetic analysis tools for polyploid become available. This review should be helpful for the scientists working on sugarcane biomass improvement through biological, genetic, and genomic approaches.

## Author Contributions

JW conceived the topic and outline and critically revised the manuscript. RK prepared the manuscript draft. XY and JS contributed critical components to the draft. All authors reviewed the manuscript.

## Conflict of Interest Statement

The authors declare that the research was conducted in the absence of any commercial or financial relationships that could be construed as a potential conflict of interest.

## Footnotes

1 http://sucest-fun.org

2 http://planttfdb.cbi.pku.edu.cn/index.php?sp=Sof

3 http://planttfdb.cbi.pku.edu.cn

4 http://grassius.org/

5 http://tropgenedb.cirad.fr/tropgene/JSP/index.jsp

6 http://www.phytozome.org

7 http://plantcellwalls.ucdavis.edu/

8 http://cellwall.genomics.purdue.edu

9 http://cell.ccrc.uga.edu/~mao/cellwall/main.htm

10 www.cazy.org

11 http://csbl.bmb.uga.edu/dbCAN/annotate.php

12 http://cys.bios.niu.edu/plantcazyme/

13 http://www.polebio.scsv.ups-tlse.fr/MAIZEWALL

14 http://wwwappli.nantes.inra.fr:8180/GTIDB/

15 http://ricephylogenomics.ucdavis.edu/cellwalls/gt/

16 http://www.personal.psu.edu/fsl/ExpCentral/index.htm
